# Elements of Inorganic Chemistry

**Published:** 1858-07

**Authors:** 


ART. X.—*Elements of Inorganic Chemistry. Including the Application of the Science in the Arts.* By THOMAS GRAHAM, F. R. S. L. & E., late Professor of Chemistry in University College, London. Edited by HENRY WATTS, B. A., F. C. S., and ROBERT BRIDGES, M. D. Second American from the Second Revised and Enlarged London Edition. Complete in one volume. Bhiladelphia: BLANCHARD & LEA. 1858.

The book before us is a ponderous volume, but none too large for the pur poses for which it is intended. It is, of course, desirable for every professional man to have a work in his library which contains every chemical fact to which he may wish at any time to refer, and such a work is the one before us. The first part of this treatise appeared some time ago, edited by Dr. Bridges, of Philadelphia, but being incomplete, was only to a certain extent valuable as a book of reference. It has now been completed under the direction of Mr. Henry Watts, making it every thing that could be desired. The illustrations are excellent and numerous, and in point of mechanical execution, it could not be improved. The entire work may be had at the low price of $4.00; Part Second, separately, with the title-page, index, &c., $2.50.

